# NMDA receptor agonists reverse impaired psychomotor and cognitive functions associated with hippocampal *Hbegf-*deficiency in mice

**DOI:** 10.1186/s13041-015-0176-0

**Published:** 2015-12-04

**Authors:** Keita Sasaki, Olaposi Idowu Omotuyi, Mutsumi Ueda, Kazuyuki Shinohara, Hiroshi Ueda

**Affiliations:** Department of Pharmacology and Therapeutic Innovation, Graduate School of Biomedical Sciences, Nagasaki University, 1-14 Bunkyo-machi, Nagasaki, 852-8521 Japan; Department of Neurobiology and Behavior, Graduate School of Biomedical Sciences, Nagasaki University, Nagasaki, 852-8523 Japan

**Keywords:** HB-EGF, Neurogenesis, NMDA receptor, Positive allosteric modulator, ADHD, OCD

## Abstract

**Background:**

Structural and functional changes of the hippocampus are correlated with psychiatric disorders and cognitive dysfunctions. Genetic deletion of heparin-binding epidermal growth factor-like growth factor (HB-EGF), which is predominantly expressed in cortex and hippocampus, also causes similar psychiatric and cognitive dysfunctions, accompanying down-regulated NMDA receptor signaling. However, little is known of such dysfunctions in hippocampus-specific *Hbegf* cKO mice.

**Results:**

We successfully developed hippocampus-specific cKO mice by crossbreeding floxed *Hbegf* and *Gng7-Cre* knock-in mice, as *Gng7* promoter-driven Cre is highly expressed in hippocampal neurons as well as striatal medium spiny neurons. In mice lacking hippocampus *Hbegf* gene, there was a decreased neurogenesis in the subgranular zone (SGZ) of the dentate gyrus as well as down-regulation of PSD-95/NMDA-receptor-NR1/NR2B subunits and related NMDA receptor signaling. Psychiatric, social-behavioral and cognitive abnormalities were also observed in hippocampal cKO mice. Interestingly, D-cycloserine and nefiracetam, positive allosteric modulators (PAMs) of NMDA receptor reversed the apparent reduction in NMDA receptor signaling and most behavioral abnormalities. Furthermore, decreased SGZ neurogenesis in hippocampal cKO mice was reversed by nefiracetam.

**Conclusions:**

The present study demonstrates that PAMs of NMDA receptor have pharmacotherapeutic potentials to reverse down-regulated NMDA receptor signaling, neuro-socio-cognitive abnormalities and decreased neurogenesis in hippocampal cKO mice.

**Electronic supplementary material:**

The online version of this article (doi:10.1186/s13041-015-0176-0) contains supplementary material, which is available to authorized users.

## Background

According to the National Comorbidity Survey Replication, more than one-quarter of adult Americans would be diagnosed with DSM-IV mental disorders based on a fully structured diagnostic interview [1]. Structural and functional changes of the hippocampus are correlated with neuropsychiatric disorders, including major depression, which is associated with a reduced hippocampal volume and consequent functional deficits [2, 3]. Hippocampal dysfunction is also closely associated with cognitive dysfunction, as seen in schizophrenia, attention-deficit hyperactivity disorder (ADHD) and Alzheimer’s disease [4–6].

Heparin-binding EGF-like growth factor (HB-EGF) is an endogenous ligand for EGF receptors, as described for ErbB1 and ErbB4 [7, 8]. Moreover, ErbB4 displays cross talk with postsynaptic density-95 (PSD-95) and NMDA receptor signaling, which are closely related to behavioral abnormalities [9, 10]. Accumulating evidence has suggested that tight coupling of the ErbB4–PSD-95–NMDA receptor complex may underlie the pathophysiological molecular signature of psychiatric and cognitive disorders [11–13]. Conditional knockout (cKO) mice lacking *Hbegf* in the ventral forebrain showed some cognitive and neuropsychiatric abnormalities [12, 13]. There are many reports of cKO mice showing psychological disorders and most of these cKO mice are deficient in specific molecules in both the cerebral cortex and hippocampus. However, very few descriptions of psychological and learning disorders in hippocampus-specific *Hbegf* cKO mice exist. The present study focused on behavioral phenotypes and therapeutic specificity in mice with a hippocampal deficiency of the *Hbegf* gene, as the hippocampus is known to be closely related to depression [2, 3], psychiatric disorders [4], cognition [5, 6] and neurogenesis [14–16]. Therefore, we developed hippocampus-specific cKO mice by crossbreeding floxed *Hbegf* [17] and *Gng7-Cre* knock-in mice [18], as *Gng7* promoter-driven Cre is highly expressed in hippocampal neurons as well as striatal medium spiny neurons, while *Hbegf* is highly expressed in cerebral cortex and hippocampal neurons [19, 20]. The resultant hippocampus-specific cKO mice retained the neuropsychiatric disorders as well as decreased learning potential, and also showed down-regulation of NMDA receptor signaling molecules. Additionally, these mice showed marked defects in neurogenesis. In the present study, we characterized the pathophysiological features of these hippocampus-specific *Hbegf* cKO mice in relation to ADHD and obsessive-compulsive disorder (OCD), and propose therapeutic avenues for inhibiting these features.

## Results

### Generation of hippocampal *Hbegf* knockout mice

**Fig. 1:**
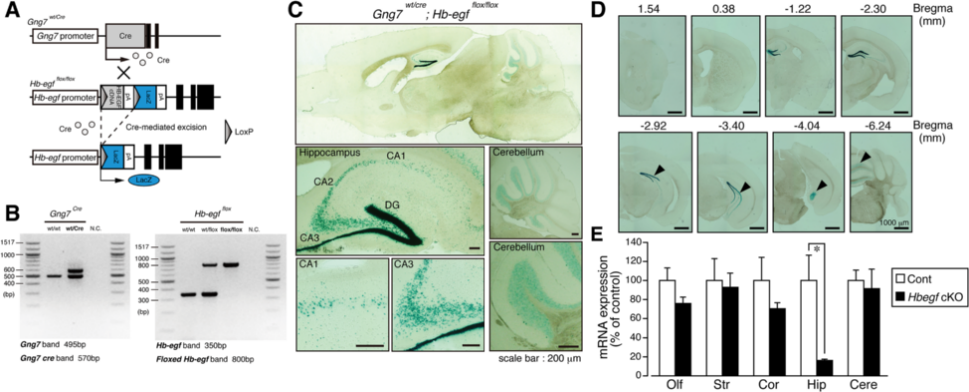
Generation and determination of restricted HB-EGF deletion in *Hbegf* cKO mice. **a** Genomic structure and the design of the *Hbegf* cKO mice. **b** Genotyping of *Hbegf* cKO mice by PCR. **c** Regional specificity of *Hbegf* deletion. LacZ staining of *Hbegf* cKO mice brain section shows specific expression of lacZ in parts of the brain including the hippocampal CA1, CA3 and dentate gyrus (DG) regions, and the cerebellar granule cell layer. **d** Brain coronal sections with LacZ staining in *Hbegf* cKO mice. LacZ positive cells are indicated by arrow heads. **e** Quantitative analysis of *Hbegf* mRNA expression in brain regions. *n* = 4 (Control), *n* = 4 (cKO). Values are the means ± s.e.m. Olf, olfactory bulb; Str, striatum; Cor, cortex; Hip, hippocampus; Cere, cerebellum

Hippocampus is the epicenter of learning and episodic memory [21], changes in the ultrastructure [22] and biochemical circuitry [23] have been implicated in neuropsychiatric disorders. Since development and maintenance of hippocampal structure and neuronal connectivity is tightly regulated by several growth factors [24], including HB-EGF [12] whose ablation in hippocampus has been linked with neuropsychiatric episodes in mice [13]. In this study, hippocampal neuron-specific *Hbegf* knockout (KO) mice was generated using floxed *Hbegf* knock-in mice [17, 25] and transgenic mice that express Cre recombinase under the control of a G protein γ subunit 7 gene (*Gng7*) promoter (Fig. 1a) [18]. These mice were crossbred and the resulting *Gng7*^*wt/cre*^; *Hbegf*^*wt/flox*^ mice were backcrossed with *Hbegf*^*flox/flox*^ mice to generate *Gng7*^*wt/cre*^; *Hbegf*^*flox/flox*^ mice. The genotypes of *Gng7*^*wt/cre*^; *Hbegf*^*flox/flox*^ mice were determined by PCR as described previously [26]. *Gng7*/*Gng7-Cre* bands (495 bp/570 bp) and floxed *Hbegf* bands (800 bp) were determined in the *Gng7*^*wt/cre*^ and *Hbegf*^*flox/flox*^ mice DNA samples (Fig. 1b) respectively.

**Fig. 2:**
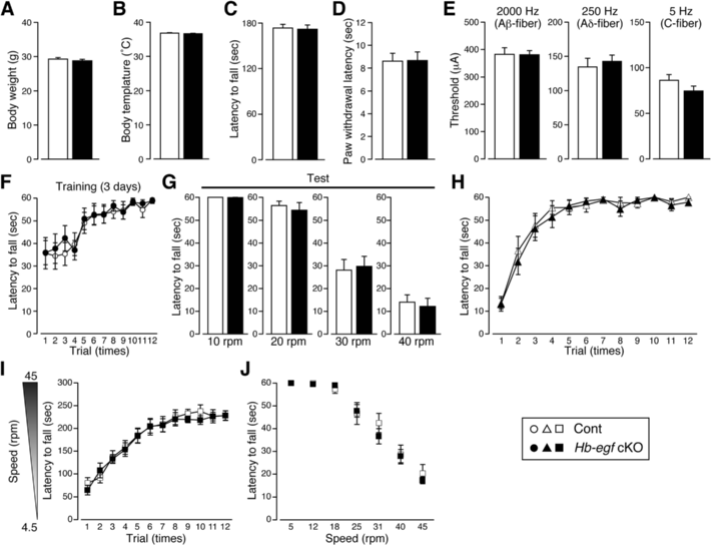
Characteristics of general health, sensory and motor function in *Hbegf* cKO mice. No significant changes in body weight: **a**, body temperature: **b**, neuromuscular strength in the wire hang test: **c**, thermal-nociceptive threshold using the thermal stimulator: **d**, or the electrical threshold of peripheral nerve (Aβ, Aδ, C-fiber) using a Neurometer CPT system: **e** were observed. No significant change in the latency to fall in the rotarod training: **f**, and test period using different speeds of 10, 20, 30, and 40 rpm: **g**, **h**, No significant change in the stationary horizontal thin-rod test. No significant change in the accelerating rotarod test in repetitive trials: **i**, and with differential speeds: **j**. During the training period, each mouse was placed on the rotarod revolving at a constant speed (4.5 rpm) and then accelerated to 45 rpm. Mice were trained for 3 consecutive days, receiving four trials per day with a 1 h inter-trial interval: (**i**). During the test period on day 4, mice were tested in seven consecutive 60 s trials at constant speeds of 5, 12, 18, 25, 31, 40 and 45 rpm. The mean latency to fall from the rotarod (for the three trials at each speed level) was recorded: (**j**). *n* = 7 (Control), *n* = 7 (cKO). All results are presented as means ± s.e.m

In order to confirm the successful hippocampal deletion of *Hbegf* using cre-lox system, LacZ staining was conducted using 30 μm brain sections from male *Gng7*^*wt/cre*^; *Hbegf*^*flox/flox*^ mice. In the adult hippocampus, LacZ-positive cells (blue) were mainly observed in hippocampal DG and CA3–CA1 layers. However, a number of cells in the cerebellar granule cell layer were also positive (Fig. 1c, d). qPCR analysis also revealed that *Hbegf* cKO mice showed a significant reduction of hippocampal (Hip) *Hbegf* mRNA, but there was no significant decrease in olfactory bulb (Olf), striatum (Str), cortex (Cor) or cerebellum (Cere), compared with control mice (Fig. 1e). Data were analyzed by Student’s *t* test (Olf: Cont: 100.00 ± 13.23 %; cKO: 75.78 ± 6.73 %; t = 1.761 df = 6, *p* = 0.1286, Str: Cont: 100.00 ± 22.96 %; cKO: 92.82 ± 14.93 %; t = 0.4295 df = 6, *p* = 0.6826, Cor: Cont: 100.00 ± 24.32 %; cKO: 70.34 ± 6.26 %; t = 1.413 df = 6, *p* = 0.6826, Hip: Cont: 100.00 ± 26.59 %; cKO: 16.13 ± 1.37 %; t = 3.492 df = 6, *p* = 0.013, Cere: Cont: 100.00 ± 10.93 %; cKO: 16.13 ± 10.53 %; t = 0.1985 df = 6, *p* = 0.849). No LacZ-signals were observed in male *Gng7-Cre* knock-in mice or floxed *Hbegf* knock-in mice (Additional file 1: Figure S1). Male hippocampal *Hbegf* cKO (*Gng7*^*wt/cre*^; *Hbegf*^*flox/flox*^) mice showed no remarkable differences in body weight (Cont: 29.24 ± 0.4610 g, *n* = 9; cKO: 28.77 ± 0.4123 g, *n* = 10; Student’s *t* test: t = 0.7696, df = 17, *p =* 0.4521), body temperature (Cont: 36.80 ± 0.1443 °C, *n* = 9; cKO: 36.64 ± 0.1310 °C, *n* = 10; Student’s *t* test: t = 0.8229, df = 17, *p =* 0.4219), wire hanging (Cont: 173.4 ± 4.811 s, *n* = 9; cKO: 171.8 ± 5.629 s, *n* = 10; Student’s *t* test: t = 0.2195, df = 17, *p =* 0.8288), thermal nociceptive threshold (Cont: 8.600 ± 0.6978 s, *n* = 9; cKO: 8.660 ± 0.7559 s, *n* = 10; Student’s *t* test: t = 0.05790, df = 17, *p =* 0.9545), and electrical stimulation-induced paw withdrawal latency (Aβ-fiber: Cont: 382.2 ± 24.25, *n* = 9; cKO: 381.0 ± 14.87, *n* = 10; Student’s *t* test: t = 0.04396, df = 17, *p =* 0.9654, Aδ-fiber: Cont: 134.4 ± 12.71, *n* = 9; cKO: 142.5 ± 9.347, *n* = 10; Student’s *t* test: t = 0.5179, df = 17, *p =* 0.6112, and C-fiber: Cont: 86.33 ± 6.410, *n* = 9; cKO: 74.70 ± 5.391, *n* = 10; Student’s *t* test: t = 1.398, df = 17, *p =* 0.18), compared with control mice (Fig. 2a–e). Furthermore, the cKO mice showed no significant motor dysfunction in terms of coordination (rotarod training: two-way repeated measures ANOVA, genotype effect, 1–12 trials, *F*_(1,17)_ = 0.057, *p* = 0.814, test period: 10 rpm: Cont: 60.00 ± 0.0, *n* = 9; cKO: 59.87 ± 0.1333, *n* = 10; Student’s *t* test: t = 0.9459, df = 17, *p =* 0.3574, 20 rpm: Cont: 56.33 ± 1.954, *n* = 9; cKO: 54.43 ± 3.220, *n* = 10; Student’s *t* test: t = 0.4906, df = 17, *p =* 0.63, 30 rpm: Cont: 28.11 ± 4.713, *n* = 9; cKO: 29.77 ± 4.427, *n* = 10; Student’s *t* test: t = 0.2562, df = 17, *p =* 0.8009, and 40 rpm: Cont: 14.04 ± 3.193, *n* = 9; cKO: 12.17 ± 3.589, *n* = 10; Student’s *t* test: t = 0.3857, df = 17, *p =* 0.7045), balance (stationary horizontal thin-rod test, two-way repeated measures ANOVA, genotype effect, 1–12 trials, *F*_(1,17)_ = 0.883, *p* = 0.360) and learning (accelerating rotarod training: two-way repeated measures ANOVA, genotype effect, 1–12 trials, *F*_(1,12)_ = 0.074, *p* = 0.790, test period: 5 rpm: Cont: 60.00 ± 0.0, *n* = 7; cKO: 60.00 ± 0.0, *n* = 7, 12 rpm: Cont: 60.00 ± 0.0, *n* = 7; cKO: 59.57 ± 0.4286, *n* = 7; Student’s *t* test: t = 1.000, df = 12, *p =* 0.337, 18 rpm: Cont: 57.29 ± 1.761, *n* = 7; cKO: 59.05 ± 0.6801, *n* = 7; Student’s *t* test: t = 0.9331, df = 12, *p =* 0.3692, 25 rpm: Cont: 46.67 ± 4.813, *n* = 7; cKO: 47.81 ± 2.796, *n* = 7; Student’s *t* test: t = 0.2053, df = 12, *p =* 0.8408, 31 rpm: Cont: 42.48 ± 4.251, *n* = 7; cKO: 36.62 ± 3.368, *n* = 7; Student’s *t* test: t = 1.080, df = 12, *p =* 0.3014, 40 rpm: Cont: 28.62 ± 4.132, *n* = 7; cKO: 27.90 ± 2.907, *n* = 7; Student’s *t* test: t = 0.1414, df = 12, *p =* 0.8899, and 45 rpm: Cont: 20.33 ± 3.895, *n* = 7; cKO: 17.33 ± 1.471, *n* = 7; Student’s *t* test: t = 0.7206, df = 12, *p =* 0.485), as shown in Fig. 2f–j.

### Reduced NMDA receptor subunit expression and altered LTP in hippocampal *Hbegf* knockout mice

**Fig. 3:**
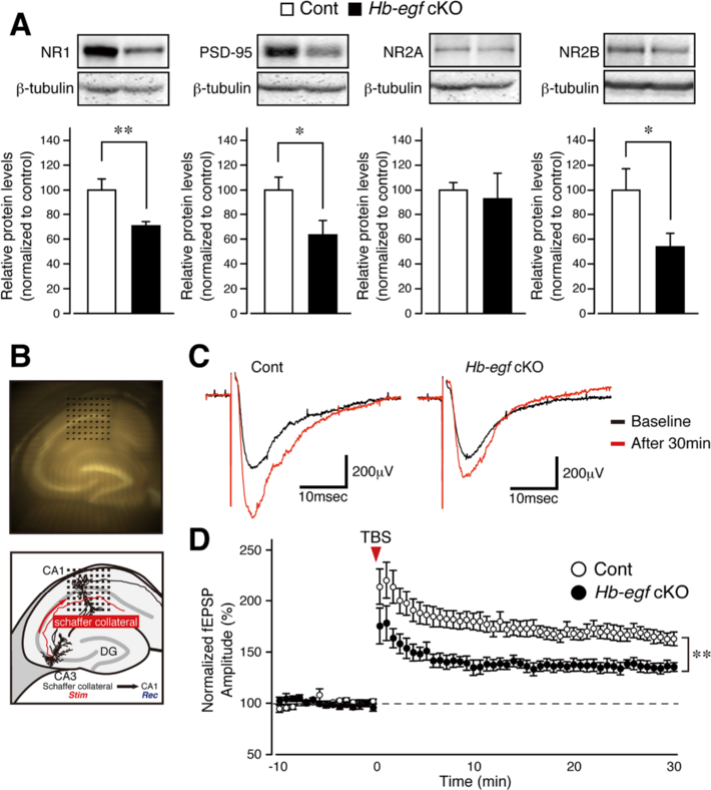
*Hbegf* deficiency decreases hippocampal NMDA-R expression and plasticity. **a**, Altered protein expression. Immunoblotting for NMDA receptor subunits (NR1, NR2A and NR2B), PSD-95 and β-tubulin in the hippocampi of control and cKO mice. *n* = 8 (Control), *n* = 8 (cKO).**b**, Micrograph and schematic illustration of a hippocampal slice placed onto the multi-electrode dish. **c**, Representative traces of fEPSPs before and after the theta burst stimulation. The fEPSPs before and 30 min after the theta burst stimuli are indicated by black and red lines, respectively. **d**, Time course of normalized fEPSP amplitude after theta burst stimulation. *n* = 9 (Control), *n* = 7 (cKO). All results are presented as means ± s.e.m

ErbB4, a bonafide receptor of HB-EGF is known to associate with postsynaptic density-95 (PSD-95) [27], PSD-95 in turn has been identified as component of the scaffold complex involved in NMDA receptor aggregation in the CNS [28] via biophysical interaction with the C-terminus of the receptor. Accumulating evidence suggest that de-coupling ErbB4–PSD-95–NMDA receptor interaction due to mutation or/and reduced expression of any of the member component may represent a key molecular signature of psychiatric episodes, cognitive disorders [29] and altered long-term potentiation (LTP). Western blot analysis performed on male *Hbegf* cKO mice showed significantly reduced hippocampal NR1/NR2B-NMDA receptor subunits and PSD-95, but not the NR2A subunit, compared with control mice (Fig. 3a). Student’s *t* test was used to compare the differences between protein levels in control and cKO mice (NR1: Cont: 100.0 ± 9.045 %, *n* = 8; cKO: 70.66 ± 3.499 %, *n* = 8; t = 3.026, df = 14, *p =* 0.0091, PSD-95: Cont: 100.0 ± 10.43 %, *n* = 8; cKO: 63.30 ± 11.71 %, *n* = 8; t = 2.340, df = 14, *p =* 0.0346, NR2A: Cont: 100.0 ± 6.226 %, *n* = 8; cKO: 92.55 ± 21.16 %, *n* = 8; t = 0.3376, df = 14, *p =* 0.7407, NR2B: Cont: 100.0 ± 17.17 %, *n* = 8; cKO: 53.81 ± 10.92 %, *n* = 8; t = 2.270, df = 14, *p =* 0.0395). To investigate resulting changes in LTP in *Hbegf* cKO mice, we performed electrophysiology experiments using acutely prepared hippocampal slices from male control and cKO mice.

Slices were placed over a multi-electrode array; a bipolar stimulation electrode was positioned on the Schaffer collaterals region, and extracellular recordings using a multi-electrode array (MED64, Alpha Med Sciences, Japan, Fig. 3b). Each field excitatory post-synaptic potential (fEPSP) in the Schaffer collateral/CA1 hippocampal pathway was adjusted to evoke a response with an amplitude of 30–40 % of the maximum population spike amplitude induced by bipolar constant current pulses (3–50 μA, 0.1 ms), through a 0.1–10 kHz bandpass filter. Each stimulation sequence consisted of a random impulse train of 200 pulses applied every 30 s. After allowing a stable baseline of 10 min, three theta burst stimuli were given to the slice, with each consisting of a ten-burst train of four pulses (100 Hz) with 200 ms inter-train intervals. The fEPSPs 30 min after the theta burst stimuli were markedly enhanced in slices from male control mice, but the enhancement was significantly decreased in slices from male cKO mice after 30 min (Fig. 3c, d). Repeated measures two-way ANOVA was used to compare the differences between amplitude levels in control and cKO mice (genotype effect, 0–30 min, *F*_(1,14)_ = 13.23, *p* = 0.003).

**Fig. 4:**
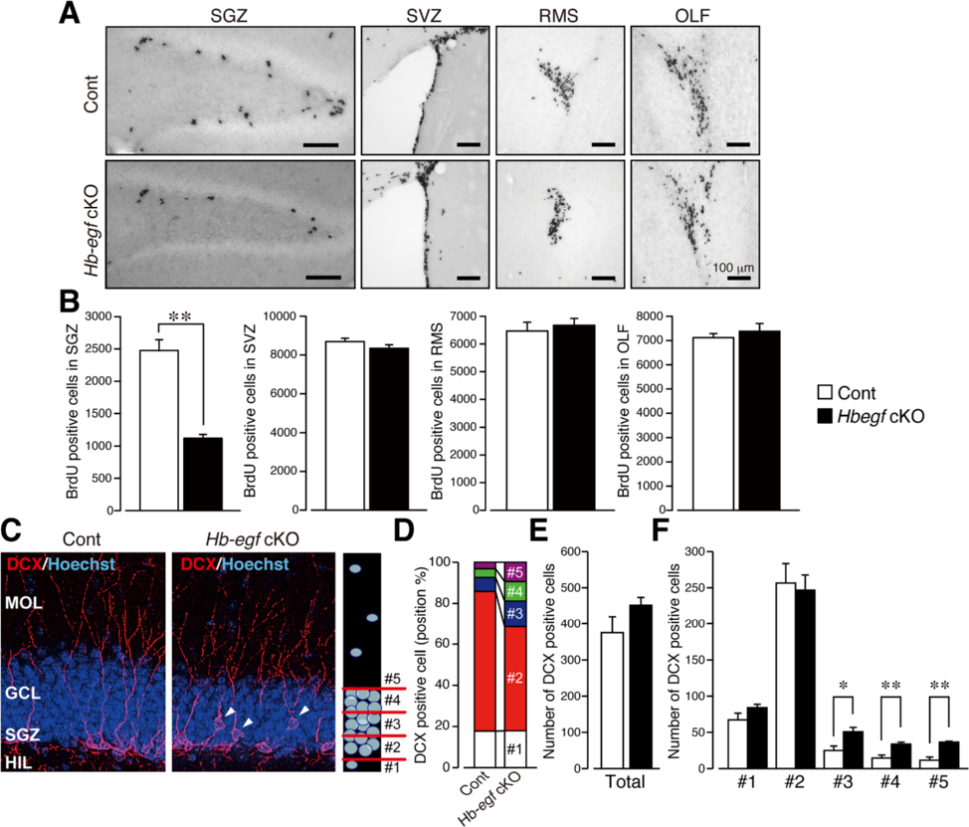
*Hbegf* deficiency aters neurogenesis in *Hbegf* cKO mice. **a** Representative pictures of BrdU-positive neurons. The specimens show BrdU-positive neurons in the subgranular zone (SGZ), subventricular zone (SVZ), rostromedial striatum (RMS) and olfactory bulb (OLF). Scale bar: 100 μm. **b** Comparison of the numbers of BrdU-positive neurons. **c** Representative pictures of detailed localization of doublecortin (DCX)-positive immature neurons in the dentate gyrus. **d** Sub-localization analysis of DCX-positive immature neurons. #1 (between of hilus and SGZ), #2 (SGZ and inner 1/3 of region of granular cell layer/GCL), #3 (medial 1/3 of GCL), #4 (outer 1/3 of GCL), #5 (molecular layer/MOL). **e**, **f** Quantitative analysis of the number of DCX-positive cells and their relative positions in dentate gyrus. **e** Total number of DCX positive cells in DG. **f** The number of DCX positive cells in each layer (#1 - #5). *n* = 4 (Control), *n* = 4 (cKO). *p <0.05 (control versus cKO). Values are the means ± s.e.m

Interestingly, some studies have demonstrated that LTP enhances neurogenesis in the adult dentate gyrus [30] and that recombinant HB-EGF administration increased the number of BrdU-positive cells in the dentate gyrus (DG) of adult mice [14]. Here, BrdU labeling of newborn neurons in the subgranular zone (SGZ) of the dentate gyrus, subventricular zone (SVZ) of the lateral ventricle, rostral migratory stream (RMS), and the olfactory bulb (OLF) was performed (Fig. 4a). As shown in Fig. 4b, male *Hbegf* cKO mice showed significant decrease in the number of BrdU positive cells in the SGZ (Cont: 2477 ± 166.7, cKO: 1148 ± 68.31, *p* = 0.0003), but not in other regions, compared with control mice (SVZ: Cont: 8693 ± 173.8; cKO: 8348 ± 179.9; t = 1.365, df = 7, *p =* 0.2145, OLF: Cont: 7122 ± 166.9; cKO: 7384 ± 320.7; t = 0.7733, df = 7, *p =* 0.4646, RMS: Cont: 6470 ± 315.0; cKO: 6836 ± 243.4; t = 0.8783, df = 7, *p =* 0.4089). *n* = 5 (Control), *n* = 4 (cKO). Given that some new neurons were formed in the *Hbegf* cKO mice, next, we sought to establish the morphological effects of *Hbegf* ablation on the developing neurons. When immature neurons were stained using an anti- doublecortin (DCX) antibody, DCX-positive cells were found in the SGZ of the DG (Fig. 4c). Most DCX-positive cells were located in the SGZ and the inner granule cell layer (GCL) of the DG (Layer 2) in male control mice. In contrast, male cKO mice showed mis-positioning of DCX-positive cells (Fig. 4d), which were found in the middle to outer layer of the GCL instead (layers #3–5), but there was no significant difference in the total number of DCX positive cells in DG (Cont: 374.8 ± 44.99; cKO: 451.9 ± 21.37; Student’s *t* test: t = 1.548, df = 6, *p =* 0.1725, Fig. 4e). Among sub regions of DG, there were significant increase in the number of positive cells in mis-positioned areas such as granular cell layer/GCL and molecular cell layer/MOL (#3-5), but not in hilus layer/HIL (#1) or subgranular zone/SGZ (#2) (layer 1: Cont: 67.25 ± 9.259; cKO: 84.13 ± 4.598; Student’s *t* test: t = 1.632, df = 6, *p =* 0.1537, layer 2: Cont: 256.5 ± 26.84; cKO: 246.8 ± 20.85; Student’s *t* test: t = 0.287, df = 6, *p =* 0.7838, layer 3: Cont: 25.00 ± 6.151; cKO: 50.75 ± 6.142; Student’s *t* test: t = 2.962, df = 6, *p =* 0.0252, layer 4: Cont: 14.50 ± 4.173; cKO: 33.75 ± 2.665; Student’s *t* test: t = 3.887, df = 6, *p =* 0.0081, layer 5: Cont: 11.50 ± 4.444; cKO: 36.50 ± 1.242; Student’s *t* test: t = 5.418, df = 6, *p =* 0.0016, Fig. 4f).

### *Hbegf* knockout mice causes dysfunctional learning behavior and impulsivity

**Fig. 5:**
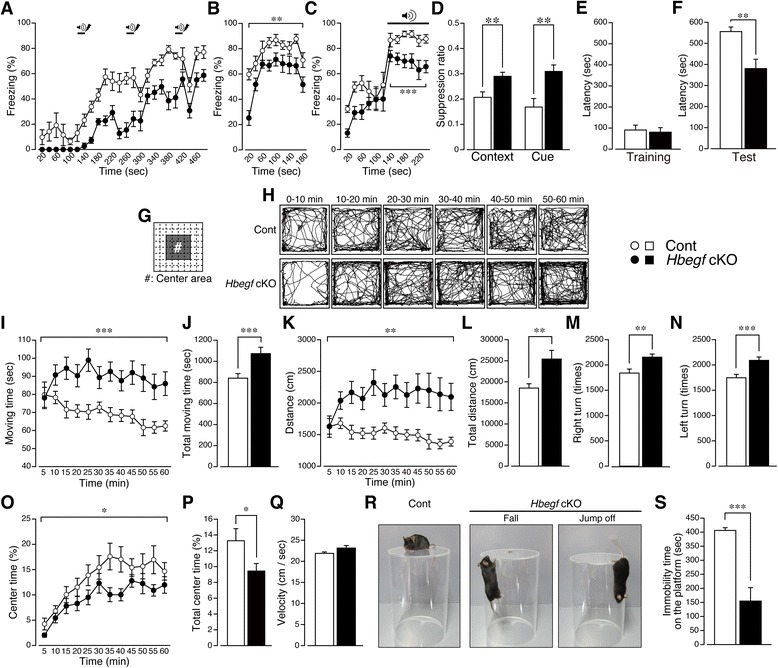
Learning and psychomotor disability in *Hbegf* cKO mice. **a**-**d**, Impaired learning and memory in the context and cued fear conditioning test. Freezing behavior was observed and shown as the percentage of freezing time. **a** For conditioning, mice were placed in a conditioning chamber and given three sets of CS and co-terminating US. *n* = 9 (Control), *n* = 10 (cKO). **b** Twenty-four hours after conditioning, contextual memory was evaluated for 3 min in the chamber used for conditioning. **c** In the cued test, mice were placed in the novel chamber for 2 min, then exposed to a CS for another 2 min. **d** Activity suppression ratio in contextual and cued testing. **e** and **f**, Impaired learning and memory in the step through type passive avoidance (PA) test. **e** In the training session, learning and memory functions in control and cKO mice were assessed based on escape latencies in the PA test. *n* = 15 (Control), *n* = 16 (cKO). **f** In the retention test 24 h after training, maximum latency was set as 600 s. **g**-**q** Hyperactivity was evaluated in Open-field test. **g** Schematic diagram showing the center area. The center area is defined as the central 4 × 4 division of the 8 × 8 division of all chamber fields, as shown in the schematic diagram (#, center area). **h** Representative moving traces in the open field (OF) test. *n* = 25 (Control), *n* = 23 (cKO). Results represent the time course of moving time every 5 min throughout 60 min: (**i**), total moving time: (**j**), moving distance every 5 min throughout 60 min: (**k**), total moving distance: (**l**), numbers of right turns: (**m**) and left turns: (**n**), time course of center time (%) every 5 min throughout 60 min: (**o**), total center time (%): (**p**), moving velocity throughout 60 min: (**q**). **r**, Typical photographs of impulsive behavior in the cliff avoidance (CA) test. Control mice normally avoid the edge of platform so as not to fall, and do not attempt to jump off (*left*). *Hbegf* cKO mice often fall (*middle*) or jump off (*right*). **s**, Immobility time on the platform in the CA test. *n* = 10 (Control), *n* = 12 (cKO). Values are the means ± s.e.m

To investigate whether disruption of the complex infers on the learning behavior in male mice, contextual and cued fear conditioning tests were performed. *Hbegf* cKO mice showed decreased basal and foot shock (with tone)-induced freezing behavior in the training session in the square chamber. Data were analyzed by repeated measures one-way ANOVA, *F*_(1, 17)_ = 65.81, *p* < 0.0001 (Fig. 5a). In the retention test after 24 h, assessing freezing behavior in the square chamber to evaluate contextual memory, there was a significant decrease in the performance of cKO mice (repeated measures one-way ANOVA: *F*_(1, 17)_ = 13.90, *p* = 0.0017, Fig. 5b). In the experiment to evaluate cue-dependent freezing behavior, mice were put in the rectangular chamber and a cue was given at 140 s after the start of the experiment, because the difference in the initial freezing between control and cKO mice disappeared at 100–140 s. Cue-dependent freezing during the period 140–200 s after the start of the experiment was significantly lower in cKO mice than in control mice (repeated measures one-way ANOVA: *F*_(1, 17)_ = 18.25, *p* = 0.0005, Fig. 5c). We therefore calculated activity suppression ratio [31, 32] in contextual testing, and also cued testing. There were significant increases in the activity suppression ratio in the contextual testing (Cont: 0.2069 ± 0.02146; cKO: 0.2899 ± 0.01623; Student’s *t* test: t = 3.121, df = 17, *p =* 0.0062), and in the cued testing (Cont: 0.1686 ± 0.03371; cKO: 0.3093 ± 0.02577; Student’s *t* test: t = 3.356, df = 17, *p =* 0.0037) (Fig. 5d). Learning/memory in *Hbegf* cKO mice was also observed in the step-through type passive avoidance (PA) test (Fig. 5e, f). The cKO mice showed a significantly decreased latency in the test schedule (Student’s *t* test: Cont: 555.7 ± 21.72 s; cKO: 380.1 ± 44.46 s; t = 3.476, df = 29, *p =* 0.0016, Fig. 5f), but not in the training session (Student’s *t* test: Cont: 90.87 ± 22.92 s; cKO: 80.50 ± 21.49 s; t = 0.3303, df = 29, *p =* 0.7435, Fig. 5e).

Next, we determined whether dysfunctional learning behavior in male *Hbegf* cKO mice has associations with anxiety and impulsivity, as tampering with chemical communication in the hippocampus is reported to interfere with motivational, emotional and cognitive processes in animals [33]. Locomotor activity was tested in the subjects using the square open-field (OF) test. The center area was defined as the dark field (#) in the square chamber, as depicted in Fig. 5g. *Hbegf* cKO mice showed increased spontaneous activity in the marginal region of the test chamber (Fig. 5h). Significant hyperactivity was observed in the time course of moving time (repeated measures one-way ANOVA: *F*_(1, 46)_ = 13.32, *p* = 0.0007, Fig. 5i), total moving time (Student’s *t* test: Cont: 834.1 ± 29.40 s; cKO: 1073 ± 60.34 s; t = 3.649, df = 46, *p =* 0.0007, Fig. 5j), moving distance every 5 min throughout 60 min (repeated measures one-way ANOVA: *F*_(1, 46)_ = 11.70, *p* = 0.0013, Fig. 5k), total moving distance (Student’s *t* test: Cont: 18126 ± 754.4 cm; cKO: 25397 ± 2061 cm; t = 3.421, df = 46, *p =* 0.0013, Fig. 5l), and numbers of right (Student’s *t* test: Cont: 1840 ± 78.60; cKO: 2155 ± 61.30; t = 3.122, df = 46, *p =* 0.0031, Fig. 5m) and left turns (Student’s *t* test: Cont: 1747 ± 67.31; cKO: 2091 ± 66.81; t = 3.621, df = 46, *p =* 0.0007, Fig. 5n). While we observed a significant decrease in center time every 5 min throughout 60 min (repeated measures one-way ANOVA: *F*_(1, 46)_ = 4.749, *p* = 0.0345, Fig. 5o) and total center time (%) (Student’s *t* test: Cont: 13.27 ± 1.442; cKO: 9.436 ± 0.9503; t = 2.179, df = 46, *p =* 0.0345, Fig. 5p), no change in moving velocity (Student’s *t* test: Cont: 21.89 ± 0.3297; cKO: 23.14 ± 0.6017; t = 1.874, df = 46, *p =* 0.0673, Fig. 5q) was recorded. These data suggest that *Hbegf* cKO mice exhibited hyperactivity and anxiety-like behavioral phenotypes as previously reported [13]. Furthermore, cliff avoidance (CA) test was used to evaluate impulsivity [34]. Most of the control mice (*n* = 12) showed approximately 1 min-long exploratory behavior on the platform of the transparent plexiglas cylinder, followed by longer staying in the center for 406.0 ± 9.893 s, while cKO mice (*n* = 10) showed a shorter latency time to fall or jump from the platform (154.8 ± 47.77 s, Student’s *t* test: *p =* 0.0001, Fig. 5r, s). There is a possibility that the shorter latency of cKO mice to fall or jump from the platform in the cliff avoidance test is attributed to their hyperactivity. However, as cKO mice always first look into the floor and fall from the platform, they unlikely fall by excessive moving. This view may be supported by the evidence that no significant change in the moving velocity of cKO mice, despite significant increases in total moving time, total distance and number of turns were observed.

### *Hbegf* cKO mice exhibit decreased social-behavioral pattern

**Fig. 6:**
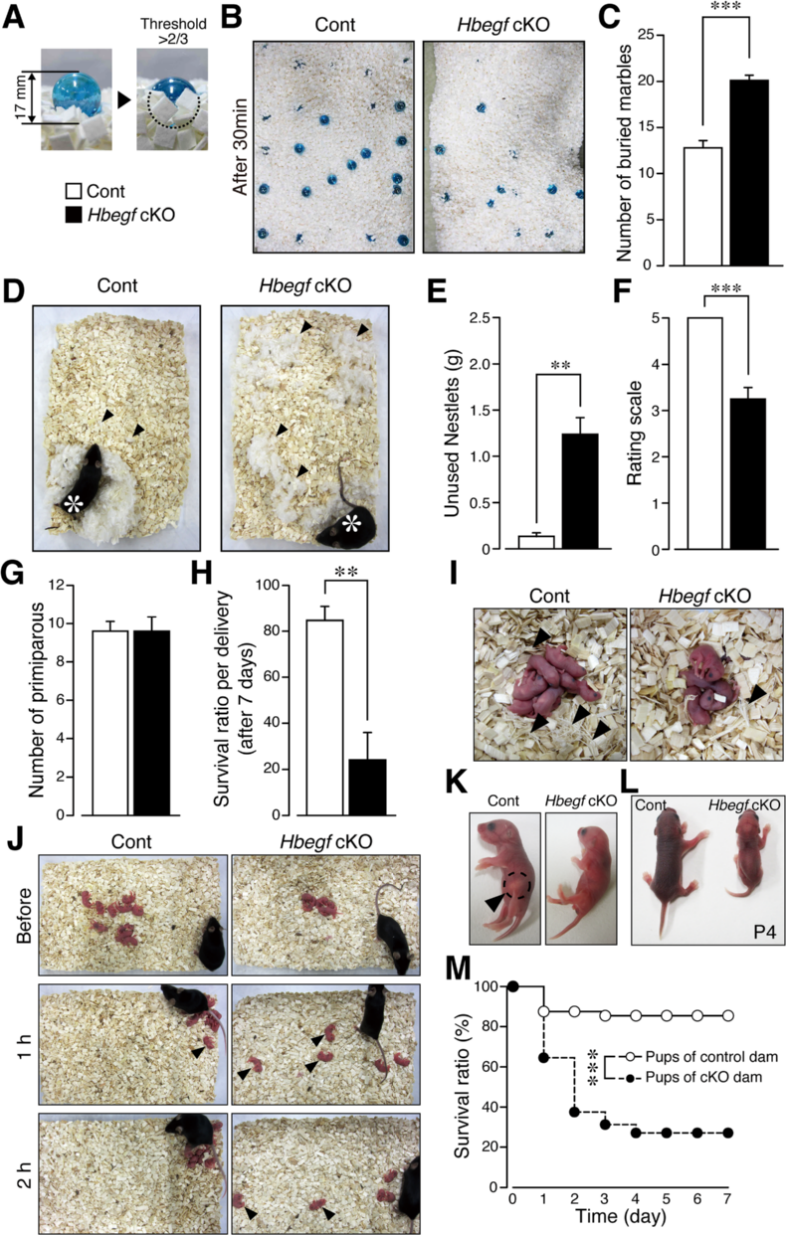
Increased obsessive behavior and decreased social activity in *Hbegf* cKO mice. **a**-**c** Obsessive behavior evaluated by the marble burying (MB) test. **a**, The burying behavior was evaluated when 2/3 of a marble was buried in the bedding. **b** Representative pictures of the results of the MB test 30 min after the start of the experiment. **c**, Quantitative comparison of MB behavior. *n* = 9 (Control), *n* = 9 (cKO). **d**-**f** Social activity as evaluated by the nest building (NB) test. **d**, Representative pictures of the nest after overnight performance in a new cage. The arrow represents the unused materials. The asterisk represents the position where the mouse sleeps. Unused Nestlet materials were determined by weight: (**e**) and the 1–5 rating scale: (**f**) *n* = 4 (Control), *n* = 4 (cKO). **g-m** Impairment of maternal behavior in female *Hbegf* cKO mice. *n* = 5 (Control dams), *n* = 5 (cKO dams). **g** Lack of change in the numbers of primiparous mice. **h** Decreased number of surviving pups at 7 days after birth. **i** Representative pictures of the nest in terms of shredding beddings (arrow). **j** Representative pictures showing the lack of maternal retrieving. **k** Lack of intragastric breast milk in neglected pups of *Hbegf* cKO mice. The arrow indicates signs of intragastric breast milk. **l** Hypotrophy in the pups of *Hbegf* cKO dam mice. Representative picture of a mouse at postnatal day 4. **m** Survival ratio. The percentage of surviving pups was evaluated over 7 days. *n* = 48 (pups of control mouse), *n* = 48 (pups of cKO dam mouse). Values are the means ± s.e.m

While hyperactivity and impulsivity are good predictors of ADHD [12, 13, 33], development of ADHD in turn negatively correlates with social interaction [35], but positively correlate with anxiety and depressive episodes [36]. The marble-burying (MB) test has been described as suitable for predicting obsessive-compulsive disorder (OCD) [37]. Interestingly, in this study, while control mice buried an average of 12.78 ± 0.7954 (*n* = 9) glass marbles out of 25 during a-30 min test, *Hbegf* cKO mice buried 20.11 ± 0.5638 (*n* = 9, Student’s *t* test: t = 7.521, df = 16, *p* < 0.0001, Fig. 6a-c). Thus, it is suggested that ablation of hippocampal *Hbegf* may confer obsessive-compulsive-like behavioral traits on the subjects. Given that increased MB activity as observed here is symptomatic of OCD, we sought to establish further that *Hbegf* cKO mice thoroughly exhibited anti-social activities using the nest building (NB) test [38]. In this test, control mice shredded the cotton plank (Nestlets®) and successfully built a nest that was taller than the height of the mouse, while cKO mice scattered the shreds of cotton without building a nest (Student’s *t* test: Cont: 0.1350 ± 0.03753 g; cKO: 1.238 ± 0.1807 g; t = 5.973, df = 6, *p =* 0.001, Fig. 6d). In quantitative analysis, higher levels of unused Nestlets (Student’s *t* test: Cont: 0.1350 ± 0.03753 g; cKO: 1.238 ± 0.1807 g; t = 5.973, df = 6, *p =* 0.001, Fig. 6e) and lower scores (Student’s *t* test: Cont: 5.000 ± 0.0; cKO: 3.250 ± 0.2500; t = 7.000, df = 6, *p =* 0.0004, Fig. 6f) on nest rating scales were observed in cKO mice. Next, maternal behavioral pattern was investigated. Although there was no significant difference in the frequencies of primiparous mice between control and *Hbegf* cKO mice (Cont: 9.600 ± 0.5099, cKO: 9.600 ± 0.7483, *p* > 0.9999, Fig. 6g), the survival ratio per delivery at day 7 after birth was markedly decreased in female *Hbegf* cKO mice (Student’s *t* test: Cont: 84.64 ± 6.248 %; cKO: 24.18 ± 11.77 %; t = 4.538, df = 8, *p =* 0.0019, Fig. 6h). When we examined the shredded bedding materials, female control animals built crater-shaped nests made of shredded bedding materials, while female *Hbegf* cKO mice did not (Fig. 6i). Furthermore, female *Hbegf* cKO mice failed to gather all 24-h-old pups (Fig. 6j), which had been put in the center of cage, into the nest (retrieving behavior) at the edge of cage at the time point of 1 h and even at 2 h in contrast to the control group. Additionally, female *Hbegf* cKO dams showed a lack of nursing (lactation behavior, Fig. 6k), and their pups showed delayed growth at postnatal day 4 (Fig. 6l) and decreased survival (log-rank test, *χ*^2^ = 27.12, ***p < 0.0001, Fig. 6m). These features suggest that female *Hbegf* cKO dams showed “pup neglect”-traits.

### NMDA mimetics reverse behavioral abnormalities in *Hbegf* cKO mice

**Fig. 7:**
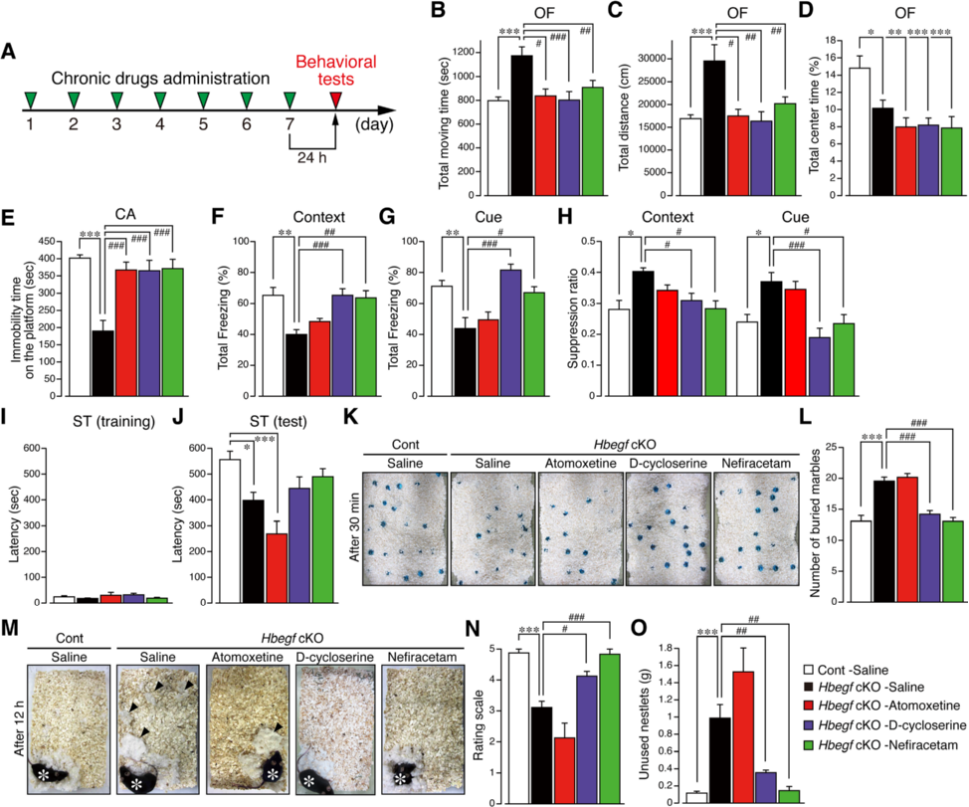
Pharmacological amelioration of behavioral dysfunctions in *Hbegf* cKO mice. **a**, Time schedule of drug treatments and behavioral tests. **b-d**, Effects of compounds on hyperactivity and anxiety in the OF test. Results are shown as moving time: (**b**), moving distance: (**c**) and center time: (**d**) in the OF test. *n* = 23, 24, 18, 15, and 14, respectively, for Control-Saline, cKO-Saline, cKO-Atomoxetine, cKO-D-cycloserine, and cKO-Nefiracetam. **e**, Effects on impulsive behavior. Immobility times on the platform (s) were evaluated in the CA test. *n* = 23, 27, 19, 18, 16, respectively, for the groups mentioned above. **f**-**h**, Effects on dysfunction in contextual-: (**f**) and cue-exposed freezing: (**g**), and activity suppression ratio: (**h**) using the fear conditioning test. All data are shown as the percent of total freezing. *n* = 8, 9, 10, 16, 10, respectively, for the groups mentioned above. **i** and **j**, Effects on memory deficit using the step-through passive avoidance (PA) test. In the training session, each chronic drug administration did not change the PA latency: (**i**). Atomoxetine further decreased the PA latency in *Hbegf* cKO mice, while the decrease induced by D-cycloserine and nefiracetam was slight, but not significant: (**j**). The numbers of animals used were 10, 14, 10, 16, and 18, respectively. **k** and **l**, Effects on the obsessive behavior in *Hbegf* cKO mice. Both D-cycloserine and nefiracetam, but not atomoxetine reversed obsessive behavior in the MB test. Representative pictures of the results of the MB test 30 min after the start of the experiment: (**k**). Quantitative comparison of the numbers of buried marbles: (**l**). The numbers of animals used were 12, 18, 18, 15, and 16, respectively. **m-o**, Effects on decreased social activity. Typical pictures of differential pharmacological effects on nest building behavior: (**m**). Quantitative comparison on the rating scale: (**n**) and the levels of unused Nestlets: (**o**). The numbers of animals used were 8, 9, 8, 16, and 6, respectively. Values are the means ± s.e.m

From the foregoing, *Hbegf* cKO mice have shown clear signs of altered social, parental, anxiety-like and locomotor behaviors. To examine whether these phenotypes are related to altered HB-EGF/ErbB4–PSD-95–NMDA receptor signaling, we tested two positive allosteric modulators (PAMs) of NMDA receptor, D-cycloserine [39] and nefiracetam [40, 41], since D-cycloserine has been reported to prevent relational memory deficits and suppression of LTP in the hippocampus [42] and nefiracetam administration is known to improve depressive behaviors [43]. In addition, we also examined the effects of atomoxetine (representative ADHD medicine), which exhibits dual norepinephrine transporter (NET) inhibition [44] and NMDA receptor antagonism [45]. All drugs were administered once daily for seven consecutive days, and behavioral tests were performed 24 h after the last administration (Fig. 7a).

In the OF test, D-cycloserine, nefiracetam and atomoxetine reversed the hyperactivity in male *Hbegf* cKO mice in terms of total moving time (ANOVA followed by a post hoc Tukey’s test: *F*_(4, 89)_ = 8.039, Fig. 7b) and total distance moved (ANOVA followed by a post hoc Tukey’s test: *F*_(4, 89)_ = 6.687, Fig. 7c). The decreased total center time, however, was not affected by any of these three compounds (ANOVA followed by a post hoc Tukey’s test: *F*_(4, 89)_ = 6.829, Fig. 7d). *p < 0.05, **p < 0.01, and ***p < 0.001 versus Control-Saline, #*p* < 0.05, ##*p* < 0.01, and ###*p* < 0.001 versus cKO-Saline. In the CA test, all three compounds reversed the impulsivity (ANOVA followed by a post hoc Tukey’s test: *F*_(4, 98)_ = 13.21, *p* < 0.001 versus Control-Saline, ###*p* < 0.001 versus cKO-Saline, Fig. 7e). In the contextual space memory-related task, both D-cycloserine and nefiracetam, but not atomoxetine significantly reversed this indication in male *Hbegf* cKO mice (ANOVA followed by a post hoc Tukey’s test: *F*_(4, 48)_ = 7.918, **P < 0.01 versus Control-Saline, ##*p* < 0.01, and ###*p* < 0.001 versus cKO-Saline, Fig. 7f). Similar results were also observed in the cue-conditioning test (ANOVA followed by a post hoc Tukey’s test: *F*_(4, 48)_ = 11.67, **p < 0.01 versus Control-Saline, #*p* < 0.05, and ###*p* < 0.001 versus cKO-Saline, Fig. 7g). In the contextual testing, increased activity suppression ratio in cKO mice was significantly reversed by D-cycloserine or nefiracetam, but not by atomoxetine (ANOVA followed by a post hoc Tukey’s test: *F*_(4, 48)_ = 4.037, *p < 0.05 versus Control-Saline, #*p* < 0.05 versus cKO-Saline). Similarly in the cue-dependent testing, increased suppression ratio in cKO mice was also significantly reversed by D-cycloserine or nefiracetam (ANOVA followed by a post hoc Tukey’s test: *F*_(4, 48)_ = 7.379, *p < 0.05 versus Control-Saline, #*p* < 0.05, and ###*p* < 0.001 versus cKO-Saline) (Fig. 7h).

In the PA test, all three compounds had no effect on latency in the training task (ANOVA: *F*_(4, 63)_ = 1.592, *p* = 0.1874, Fig. 7i). At the test session, atomoxetine further shortened the latency, suggesting this action is independent of the inhibition of hyperactivity (ANOVA followed by a post hoc Tukey’s test: *F*_(4, 63)_ = 6.511, *p < 0.05 and ***p < 0.001 versus Control-Saline, Fig. 7j), on the other hand, D-cycloserine and nefiracetam showed an insignificant tendency to reverse the decreased latency in male cKO mice. In the MB test, both D-cycloserine and nefiracetam but not atomoxetine reversed this abnormal behavior in male *Hbegf* cKO mice (ANOVA followed by a post hoc Tukey’s test: *F*_(4, 74)_ = 29.24, ***p < 0.001 versus Control-Saline, ###*p* < 0.001 versus cKO-Saline, Fig. 7k, l). In the NB test, D-cycloserine and nefiracetam abolished the abnormal behavior in terms of the levels of nest rating (ANOVA followed by a post hoc Tukey’s test: *F*_(4, 42)_ = 19.92) and unused Nestlets (ANOVA followed by a post hoc Tukey’s test: *F*_(4, 42)_ = 20.09), whereas atomoxetine worsened this behavior (Fig. 7m-o). ***P < 0.01 versus Control-Saline, #*p* < 0.05, ##*p* < 0.01, and ###*p* < 0.001 versus cKO-Saline. From the foregoing, atomoxetine had no effect on abnormal MB activity whilst worsening nest-building activity. In contrast, D-cycloserine and nefiracetam significantly reversed these abnormal behaviors, thus, indicating that the pathophysiological mechanisms and pharmacological actions in the MB and NB tests appear to be independent of hyperactivity and its regulation.

### Neurobiology of therapeutic actions

**Fig. 8:**
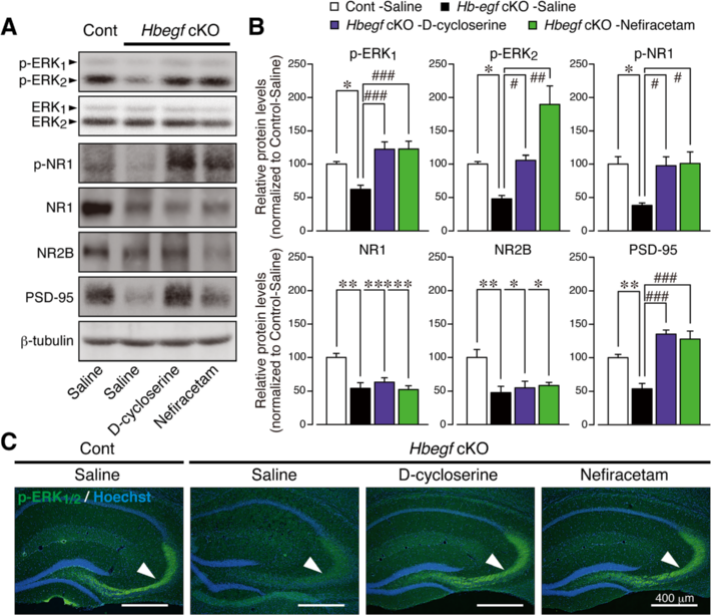
Neurobiological effects of D-cycloserine and nefiracetam in *Hbegf* cKO mice. **a-c**, Effects of D-cycloserine and nefiracetam on the decrease in levels of p-ERK_1_, p-ERK_2_, p-NR1, total NR1, NR2B and PSD-95. Schedule of drug treatments were the same as in Fig. 6a. Results represent typical pictures of western blot analysis: (**a**), and quantitative comparisons: (**b**). *n* = 5 (all groups). Values are the means ± s.e.m. Typical pERK_1/2_-like immunohistochemical pictures in the hippocampus: (**c**)

We have demonstrated that the alteration in social, parental, and locomotor behaviors and anxiety-like phenotypes in male *Hbegf* cKO mice has links with changes in neurobiological assembly of PSD-95 [27]/NMDA-receptor NR2 subunit [28] in the hippocampus. Next, we sought to examine whether the observed reversal of behavioral phenotypes in *Hbegf* cKO mice by D-cycloserine and nefiracetam has direct implication on the ErbB/PSD-95/NMDA receptor signaling. Indeed, hippocampus samples derived from male *Hbegf* cKO mice showed a marked decrease in the levels of phosphorylation of ERK_1/2_ and NR1, and in the protein levels of NR1, NR2B and PSD-95 (Fig. 8a). As shown in Fig. 8b, both D-cycloserine and nefiracetam [46] significantly increased the phosphorylation level of ERK_1_, ERK_2_ and NR1 over saline-treated *Hbegf* cKO group (ANOVA followed by a post hoc Tukey’s test: p-ERK_1_: *F*_(3, 16)_ = 10.62, p-ERK_2_: *F*_(3, 16)_ = 15.91, *p* < 0.0001, p-NR1: *F*_(3, 16)_ = 6.019, total NR1: *F*_(3, 16)_ = 10.64, NR2B: *F*_(3, 16)_ = 6.323, and PSD-95: *F*_(3, 16)_ = 20.08, * *p* < 0.05, ** *p* < 0.01, and *** *p* < 0.001 versus Control-Saline, # *p* < 0.05, ## *p* < 0.01, ### *p* < 0.001 versus cKO-Saline). Similarly, the treatments increased PSD-95 expression but not NR1 and NR2B over the saline-treated male *Hbegf* cKO group. Immunohistochemical analysis revealed that both D-cycloserine and nefiracetam mainly reversed the decrease in phosphorylation of ERK_1/2_ at the level of mossy fiber of the hippocampus (Fig. 8c).

**Fig. 9:**
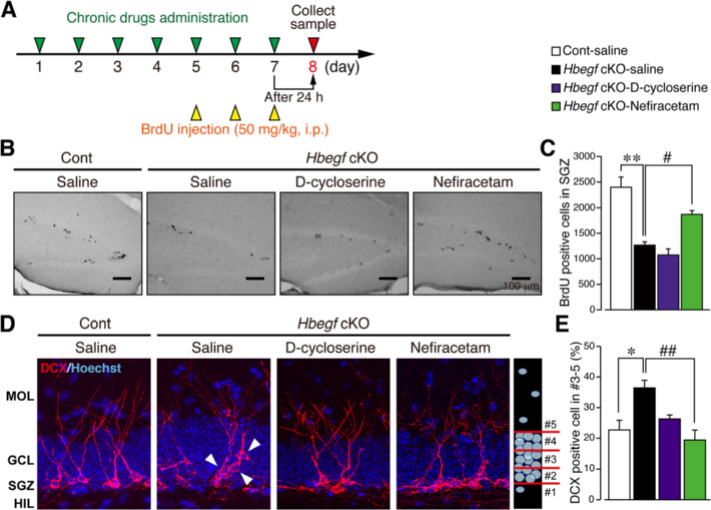
Improvements in decreased neurogenesis and abnormal sub-localization of DCX-positive neurons in *Hbegf* cKO mice. **a**, Time schedule for drug treatments and sample preparation. **b** and **c**, Representative pictures of BrdU-staining following D-cycloserine or nefiracetam-treatments: (**b**) and quantitative comparisons: (**c**). *n* = 4 (all groups). **p < 0.01 versus Control-Saline, #*p* < 0.05 versus cKO-Saline. Values are the means ± s.e.m. **d**, Representative pictures of DCX-staining following D-cycloserine or nefiracetam-treatments. **e**, Effects of D-cycloserine and nefiracetam on mis-positioning of DCX-positive neurons in GCL and MOL of DG of cKO mice. Results were represented as % ratio of mis-positioned DCX-positive neurons (#3-5 layers) to total number of cells in each slice. *p < 0.05 (control-saline versus cKO-saline), ##*p* < 0.05 (cKO-saline versus cKO-nefiracetam). The numbers of animals used were 5, 5, 4, and 5, respectively. Values are the means ± s.e.m

Chronic treatments with nefiracetam, but not D-cycloserine (Fig. 9a), reversed the decrease in neurogenesis in terms of the numbers of BrdU-positive cells in the SGZ of male *Hbegf* cKO mice (Fig. 9b, c). Data were analyzed with ANOVA followed by a post hoc Tukey’s test: *F*_(3, 12)_ = 22.59, **p < 0.01 versus Control-Saline, #*p* < 0.05 versus cKO-Saline. Moreover, the abnormal appearance of DCX-positive cells in the middle to outer layer of the GCL instead (layers 3–5) of male *Hbegf* cKO mice was significantly and partially reversed by nefiracetam and D-cycloserine, respectively (ANOVA followed by a post hoc Tukey’s test: *F*_(3, 15)_ = 7.322, *p < 0.05 versus Control-Saline, ##*p* < 0.01 versus cKO-Saline, Fig. 9d, e).

## Discussion

Previous study [13] has reported that there was a psychiatric disorder in conditional *Hbegf* KO mice using *Six3-Cre* mice, whose gene expression is widely distributed in retina and ventral forebrain including basal ganglia, medial amygdaloid area, hypothalamus, olfactory bulb and septum [47]. The ventral forebrain cKO mice showing *Hbegf* gene disruption in prefrontal cortex and hippocampus manifest hyperactivity and decreased pre-pulse inhibition, which were reversed by typical and atypical antipsychotics in behavioral studies [13]. In addition, the ventral forebrain cKO mice showed disorders in social activity and working memory, which is related to forebrain dysfunction. The mice also showed decreased levels of NMDA receptor-related signaling only in the prefrontal cortex, though it remained elusive whether these changes occur in the hippocampus. In the present study, we successfully generated hippocampal *Hbegf* cKO mice, by crossbreeding floxed *Hbegf* and *Gng7-Cre* knock-in mice. The specificity was observed in the transcription study, which shows a selective decrease of *Hbegf* gene expression in hippocampus, but not in olfactory bulb, striatum, cortex or cerebellum.

To characterize the phenotypes of hippocampal cKO mice we performed various cellular and molecular biological studies. First, we examined the NMDA receptor signaling, which is augmented through ErbB4 receptor, the target for HB-EGF [7]. Significant decrease was observed in the protein expression of NR1, NR2B and PSD-95, but not NR2A in the hippocampus, being in contrast to the case with ventral forebrain *Hbegf* cKO mice, where the decrease was observed only in NR1 and PSD-95, but not NR2A or NR2B in the prefrontal cortex [13]. Hippocampal *Hbegf* cKO mice also showed significant decreases in the phosphorylation levels of ERK_1/2_, NR1, which are closely associated to the NMDA signaling. Lack of *Hbegf* gene in hippocampal neurons induced a reduced LTP based on Schaffer collateral-CA1 pathway, which is closely related to NMDA receptor functions, in the experiment using multi-electrode arrays. However, by using this apparatus we could not detect the LTP based on perforant path-DG and mossy fiber-CA3 pathways, which are related to NMDA receptor and metabotropic glutamate receptors function, respectively [48–50]. Known electrophysiological studies using microelectrodes would be necessary to see LTP based on these pathways as the next subjects. It should be noted that hippocampal cKO mice showed a significant reduction in neurogenesis evaluated by BrdU-labelling only in the SGZ of DG, but not in the SVZ, RMS or OLF, compared with control mice. Furthermore DG of hippocampal cKO mice also showed a mis-positioning of immature neurons stained with DCX, being consistent with the previous report that HB-EGF plays roles in the maturation of neurons as a growth factor, and enhances neurogenesis in the SGZ [14].

In the behavioral studies, the cKO mice showed a dysfunction in learning behavior in the contextual and cued fear conditioning tests, where the hippocampus plays key roles. The *Hbegf* cKO mice showed significant decreases in baseline freezing without a foot shock or sound, and in freezing in the contextual task after training. An important argument arises that lower basal freezing level in hippocampal cKO mice is responsible for the decreased retention activity in fear conditioning test. However, the increase of activity suppression ratio in the contextual and cue-dependent testing was both significant after normalization. Furthermore, as these mice did not show any significant difference in the EPW test, the possibility that cKO mice are less responsive to electrical footshock would not be supported. Taking into account that passive avoidance activity in the PA test was decreased in hippocampal cKO mice, they apparently manifest the loss of learning activity. Significant decrease in the total center time may indicate that cKO mice show anxiety-like behavioral phenotype. However, the possibility cannot be excluded that hyperactivity may affect this phenotype. Impulsivity or the reduction of immobility time due to fall or jump does not seem to be simply attributed to the hyperactivity, since no significant change in the velocity in the OF test was observed and cKO mice always first look into the floor before falling from the platform. Thus, the cKO mice appear to show both hyperactivity and impulsivity, which are major symptoms observed in ADHD patients, along with cognitive dysfunction [51].

OCD, characterized by obsessive symptoms, on the other hand, was also demonstrated in the cKO mice. In the present study, the cKO mice showed excessive marble burying activity and lower nest-building ability, which represent obsessive-like behavior and social activity, respectively, for which normal hippocampal function is required [52–54]. As these abnormalities were not improved by atomoxetine, which inhibited hyperactivity and impulsivity, excessive marble burying activity and lower nest-building ability may not be attributed to hyperactivity. Additionally, the cKO mice also showed lower levels of maternal behaviors, such as retrieving and nursing (lactation behavior), which is defined as “pup neglect”.

Tourette syndrome is a neurodevelopmental condition characterized by multiple motor and vocal tics, often accompanied by behavioral symptoms, which may manifest as complex clinical features [55]. Large clinical studies indicate that many Tourette syndrome patients (estimated to be ~60 %), have a psychiatric comorbidity such as ADHD and OCD [56]. Although the assessment of vocal and motor tics is important, *Hbegf* cKO mice may manifest comorbid psychiatric features of Tourette syndrome. ADHD, OCD and Tourette syndrome are considered to be developmental diseases, which often appear in childhood [57, 58]. As the levels of HB-EGF are highly expressed in neurons in the embryonic and neonatal rodent brain, followed by a gradual decline with age in *in situ* studies [20], low HB-EGF levels in an early developmental stage may play key roles as a cause of psychiatric disorders. The findings that HB-EGF plays roles in the maturation of neurons as a growth factor, and enhances neurogenesis in the SGZ [12, 14], suggest possible roles for this factor in normal psychiatric development and learning activity.

Social withdrawal, cognitive and psychiatric abnormalities exhibited by *Hbegf* cKO mice are similar to those observed in mice lacking neurexin-1α gene (well characterized in autism and schizophrenia) [59], synaptosomal-associated protein 25 (SNAP-25) gene (Coloboma mouse mutant) [60] and dopamine active transporter (DAT) KO mice (animal model of ADHD) [61]. This study provided evidence suggesting that psychiatric abnormalities in *Hbegf* cKO mice may be related to the alteration in HB-EGF/ErbB4–PSD-95–NMDA receptor signaling, since HB-EGF is known to activate NMDA receptors [62] concentrated and anchored at the synapse via PSD-95 scaffold [28] and PAMs of NMDA receptor such as D-cycloserine [39] and nefiracetam [40, 41] aptly reversed NMDA receptor signaling modulation and all behavioral abnormalities tested except in the OF test. Specificity was observed in the finding that representative ADHD medicine atomoxetine inhibited only hyperactivity and impulsivity, but not learning/memory or social behavioral abnormalities. These pharmacotherapeutic differences support the view that learning/memory, impulsivity or social abnormalities are not simply attributed to the hyperactivity of cKO mice. Both PAMs significantly reversed NMDA receptor signaling, such as decreased phosphorylation of ERK_1/2_, NR1 and down-regulation of NR1, NR2B and PSD-95, though it remains elusive whether these pharmacological actions underlie the therapeutic actions against behavioral abnormalities. As PAMs markedly reversed the decreased ERK_1/2_ phosphorylation in the mossy fiber of cKO mice, further pharmacological studies on LTP and NMDA receptor signaling using sub regions of hippocampus should be necessary as the next subjects.

D-cycloserine is known to bind the glycine-binding site on the NMDA receptor NR1 subunit [63], and to play a partial agonist role [39, 42]. The choice of D-cycloserine was informed by previous findings that this compound showed preclinical benefits in Neuroligin 1 KO mice (human autism and mental retardation) [64] and Tourette syndrome with accompanying ADHD and/or OCD [65]. The present study revealed that D-cycloserine significantly inhibited the ADHD- and OCD-like behavioral phenotypes as well as the learning disabilities in *Hbegf* cKO mice. Similar results were observed with nefiracetam, another PAM of NMDA receptors via the glycine-binding site [40, 41, 46]. Recently we have clarified the mode of nefiracetam binding to NR1 subunit by a molecular dynamics simulation study, in which nefiracetam first binds to a novel site of NR1 and causes a release of glycine, followed by the direct binding (replacement) to the glycine pocket [41].

The reduction in SGZ neurogenesis was selectively reversed by nefiracetam, but not by D-cycloserine. The mechanisms underlying the nefiracetam-selective reversal may be in part attributed to the differential modes of action to NR1, as above-mentioned. However, the possibility of non-NMDA receptor-based mechanisms cannot be excluded, and there is a report that nefiracetam increases the number of immature neurons expressing polysialylated neural cell adhesion molecule (PSA-NCAM) in the DG, possibly through an action on nicotinic acetylcholine receptors [66]. Nevertheless, nefiracetam significantly, and D-cycloserine partially reversed the abnormal expression of DCX-positive neurons in the SGZ and DG of *Hbegf* cKO mice, where some DCX-positive neurons were observed at the inner part of DG, suggesting both PAMs reverse the abnormal maturation of newly born neurons. The further studies are necessary whether these pharmacological actions are mediated though NMDA receptor signaling or whether pharmacologically improved neurogenesis and neuronal maturation are involved in the pharmacotherapeutic actions against behavioral abnormalities.

## Conclusions

In the present study, we demonstrate that the hippocampal *Hbegf* cKO mouse is a unique model manifesting experimental ADHD/OCD-like behavioral phenotypes. Additionally, PAM of NMDA receptor would be one of promising candidates to cure related pathologies.

## Methods

### Generation of hippocampal neuron-specific *Hbegf*-deficient mice (*Gng7*^*wt/cre*^*; Hbegf*^*flox/flox*^)

All experimental protocols were approved by the Nagasaki University Animal Care Committee (Nagasaki, Japan), and were performed in accordance with approved guidelines and regulations. We generated a mutant strain of hippocampal neuron-specific knockout (KO) mice using a Cre-lox-mediated conditional *Hbegf* KO approach with the *Gng7* promoter. The generation of floxed *Hbegf* knock-in mice [17] and *Gng7-Cre* knock-in mice [18] have been described previously. Regarding the genetic background of mice, the sub strain is C57BL/6J and mice were backcrossed onto a C57BL/6J line for at least ten generations. *Hbegf*^*flox/flox*^ mice were obtained by interbreeding the *Hbegf*^*wt/flox*^ mice. *Hbegf*^*flox/flox*^ mice were bred with *Gng7-Cre* knock-in mice to generate *Gng7*^*wt/cre*^*; Hbegf*^*wt/flox*^ mice. Subsequently, *Gng7*^*wt/cre*^*; Hbegf*^*wt/flox*^ mice were bred with *Hbegf*^*flox/flox*^ mice to generate *Gng7*^*wt/cre*^*; Hbegf*^*flox/flox*^ mice (*Hbegf* cKO mice). We used male mice carrying the *Gng7-Cre* transgene for breeding to avoid an influence on maternal behavior by genetic modifications. The *Gng7-Cre* allele was identified by PCR using the primers 5′-GGCGACGTTGTTAGTACCTGAC-3′, 5′-ATCCCTGAACATGTCCATCAGGTTC-3′ and 5′-TATAGGTACCCAGAAGTGAATTCGGTTCGC-3′. Also, the floxed *Hbegf* knock-in allele was identified by PCR using the primers 5′-ATGGGATCGGCCATTGAACA-3′, 5′-GAAGAACTCGTCAAGAAGGC-3′, 5′-AGGGCAAGATCATGTGTCCTGCCTCCAGCC-3′ and 5′-CATGATGCTCCAGTGAGTAGGCTCTGATTAC-3′.

It would be highly desirable that *Gng7*^*wt/wt*^*; Hbegf*^*flox/flox*^, *Gng7*^*wt/cre*^*; Hbegf*^*wt/wt*^, and *Gng7*^*wt/wt*^*; Hbegf*^*wt/wt*^ are used as the littermate control of *Gng7*^*wt/cre*^*; Hbegf*^*flox/flox*^ (*Hbegf* cKO) mice. Due to the practical difficulty of use of all these littermate controls, male *Gng7*^*wt/cre*^ mice were used as controls for male *Hbegf* cKO mice to rule out a possible influence of genetic background, since former mice showed no significant change in psychomotor activity, compared with wild-type mice [26].

All mice were used at 10–16 weeks of age and kept in a room with a temperature of 21 ± 2 °C with *ad libitum* access to a standard laboratory diet and tap water in standard animal cages in a 12-h light/dark cycle (lights on at 08:00).

### Quantitative real-time polymerase chain reaction (qPCR)

Total RNA was extracted from each brain regions; olfactory bulb, striatum, cortex, hippocampus, and cerebellum using RNAeasy micro kit (QIAGEN, Hilden, Germany), and 500 ng of RNA was used for cDNA synthesis. qPCR was performed with GeneAce SYBR qPCR mixα (NIPPON GENE, Tokyo, Japan) using the LightCycler 480 Instrument II (Roche Diagnostics, Basel, Switzerland). The following primers were used: Glyceraldehyde-3-phosphate dehydrogenase (GAPDH), 5′-TATGACTCCACTCACGGCAAAT-3′ (forward) and 5′-GGGTCTCGCTCCTGGAAGAT-3′ (reverse) *Hbegf*, 5′- CGGGGAGTGCAGATACCTG-3′ (forward) and 5′- TTCTCCACTGGTAGAGTCAGC −3′ (reverse). Samples were amplified for 50 cycles consisting of 95 °C (15 s), 60 °C (1 min). GAPDH was used as an internal control for normalization. In all cases, the validity of amplification was confirmed by the presence of a single peak in the melting temperature analysis and by linear amplification with increasing number of PCR cycles.

### Western blot analysis

The hippocampus was sonicated in ice-cold SDS lysis buffer (50 mM Tris–HCl, pH6.8, 2 % SDS, 10 % glycerol, 1 μM [p-amidinophenyl] methanesulfonyl fluoride hydrochloride [p-APMSF]). Total protein (20 μg) was separated on SDS-polyacrylamide gels. Primary antibodies were used after following dilutions: mouse anti-NR1 antibody (1:500; Upstate, Lake Placid, NY, USA), rabbit anti-phospho-NR1 antibody (1:500; Upstate), rabbit anti-NR2A antibody (1:500; Millipore, Billerica, MA, USA), rabbit anti-NR2B antibody (1:500; Chemicon, Temecula, CA, USA), rabbit anti-PSD-95 antibody (1:500; Chemicon), rabbit anti-p44/42 MAPK (ERK_1/2_) polyclonal antibody (1:1000; Cell Signaling Technology, Danvers, MA, USA), rabbit anti-phospho-p44/42 MAPK (p-ERK_1/2_) polyclonal antibody (1:1000; Cell Signaling Technology), and rabbit anti-β-tubulin polyclonal antibody (1:500; Santa Cruz Biotechnology, Dallas, TX, USA). Horseradish peroxidase-labeled anti-mouse IgG, and horseradish peroxidase-labeled anti-rabbit IgG were used as secondary antibodies at a dilution of 1:2000 (Promega, Fichburg, WI, USA). Immunoreactive bands were detected using an enhanced chemiluminescent substrate (SuperSignal West Pico or Dura Chemiluminescent Substrate; Pierce Chemical, Dallas, TX, USA). The relative densities of the pERK_1/2_ bands were normalized to total ERK_1/2_, while those of NMDA receptor subunit bands were normalized to β-tubulin.

### Histology and immunohistochemistry

Under deep pentobarbital anaesthesia (50 mg/kg, i.p.), male mice were perfused transcardially with 20 ml of potassium-free PBS (K^+^-free PBS, pH 7.4), followed by 50 ml of a 4 % paraformaldehyde (PFA) solution in potassium-free PBS. Brains were isolated, post-fixed for 3 h, and cryoprotected overnight in a 25 % sucrose solution. Tissues were fast frozen in cryoembedding compound in a mixture of ethanol and dry ice and stored at −80 °C until use. Brain tissue was cut on a cryostat at a thickness of 30 μm, and then brain sections were thawed into the 0.1 % sodium azide stock solution at 4 °C until use. β-Galactosidase (lacZ) staining was conducted overnight at 37 °C in PBS containing 5 mM potassium hexacyanoferrate (III), 5 mM potassium hexacyanoferrate (II), 2 mM magnesium chloride and 1 mg/ml 5-bromo-4-chloro-3-indolyl-β-D-galactopyranoside. Immunohistochemistry was conducted using brain sections that were washed with TBST (0.1 % Triton X-100 in TBS). The sections were incubated with blocking buffer containing 3 % bovine serum albumin in TBST and subsequently reacted with rabbit anti-phospho-ERK_1/2_ (1:300) overnight at 4 °C. After washing, the sections were incubated with second antibody, Alexa 488-conjugated anti-rabbit IgG (1:300; Invitrogen, CA, USA) for 2 h at room temperature. After further washing, the sections were mounted with Pristine Mount, and analysed using a structured illumination microscopy (BZ-X700, Keyence, Osaka, Japan).

### Electrophysiological recordings

Brains were rapidly removed from male mice and the hippocampus region was dissected out. Transverse hippocampal slices (350 μm thick) prepared using a vibratome were incubated for 2 h in artificial cerebrospinal fluid (ACSF) containing the following (in mM): 124 NaCl, 3 KCl, 26 NaHCO_3_, 2 CaCl_2_/2H_2_O, 1 MgSO_4_/7 H_2_O, 1.25 KH_2_PO_4_, 10 D-Glucose, pH 7.2–7.4, bubbled with carbogen gas (95 % O_2_, 5 % CO_2_) at room temperature. After 2 h, a slice was transferred to 8 × 8 planar multielectrode arrays (electrode size, 20 μm × 20 μm; interpolar distance, 100 μm; Panasonic MED-P210A), and maintained submerged condition at 32 °C, while superfused at 6 ml/min (using a peristaltic pump) with ACSF used for storage, bubbled with carbogen gas (95 % O_2_, 5 % CO_2_). For recordings, extracellular field excitatory post-synaptic potentials (fEPSPs) in the Schaffer collateral/CA1 hippocampal pathway (SC/CA1) synaptically evoked fEPSPs with a magnitude of 30–40 % of the maximum response at bipolar constant current pulses (3–50 μA, 0.1 ms) and were recorded through a 0.1–10 kHz bandpass filter using a multielectrode recording system (MED64, Alpha Med Sciences) [67]. Before and after the induction of long-term potentiation (LTP), test and control pathways were stimulated every 30 s. After achieving a stable baseline for 10 min, LTP was induced by three trains of theta burst stimulation, with each theta burst consisting of a ten-burst train of four pulses (100 Hz) with 200 ms inter-train intervals. After the theta burst stimulation, fEPSPs were recorded for at least 30 min.

### Design of behavioral experiments

In all experiments, the order of behavioral tests was designed according to the expected degree of stress induced. To avoid cross-test interaction, each test was performed after a habituation period of 1 week. Only one behavioral test was performed each day. Furthermore, the same male mice were used for the following tests; assessment of general health, OF test, rotarod test, stationary horizontal thin-rod test, CA test, MB test, NB test, contextual and cued fear conditioning test, and PA test. In the initial behavioral battery experiments, we started with *n* = 6 per each group and performed the statistical comparison between control and *Hbegf* cKO mice. To clarify the reliable significance of the difference, we performed additional experiments in some tests. In this case we confirmed that there is no significant difference in respective data between the initial and additional mice, which have been used over a week after arrival. A different group of female mice was used for the tests for parity and maternal behavior. Also, independent groups of male mice were used for the experiments to determine the effects of drugs.

### Assessment of general health

Body weight and rectal temperature (as body temperature) of 10–16-week-old male control and *Hbegf* cKO mice were measured. In the wire hang test, the male mouse was first placed on a 1 × 1 cm wire mesh. The wire mesh was then inverted and waved gently, so that the mouse gripped the wire. Latency to fall was recorded, with a 180 s cutoff time. The nociception threshold was evaluated by the latency of paw withdrawal upon a thermal stimulus, using a thermal stimulator (IITC Life Science, CA, USA), as described previously [68]. A cutoff time of 20 s was set to avoid tissue damage. The electrical stimulation-induced paw withdrawal (EPW) test was performed as described previously [68, 69]. Briefly, electrodes were fastened to the plantar surfaces and insteps of male mice. Transcutaneous nerve stimuli with each of the three sine-wave pulses (5, 250, and 2000 Hz) were applied using a Neurometer® CPT® system (Neurotron, MD, USA). The minimum intensity (μA) at which each mouse withdrew its paw was defined as the current stimulus threshold.

### Rotarod test

The rotarod test was performed as previously reported [26]. The rotarod apparatus (MK0610A, Muromachi KIKAI) was used to measure fore- and hind-limb motor coordination. During the training period, each male mouse was placed on the rotarod revolving at a constant speed (20 rpm) for a maximum of 60 s, and the latency to fall off the rotarod within this time period was recorded. Mice were trained for 3 consecutive days, receiving four trials per day with a 1 h inter-trial interval. During the test period on day 4, mice were tested in four consecutive 60 s trials at constant speeds of 10, 20, 30 and 40 rpm. The mean latency to fall from the rotarod (for the four trials at each speed level) was recorded.

### Accelerating rotarod test

The accelerating rotarod test was used to measure motor learning as previously reported [70]. During the training period, each male mouse was placed on the rotarod at a constant speed (4.5 rpm), which was accelerated to 45 rpm. The maximum observation time was 5 min. Mice were trained for 3 consecutive days, receiving four trials per day with a 1 h inter-trial interval. During the test period on day 4, mice were tested for seven consecutive 60 s trials at constant speeds of 5, 12, 18, 25, 31, 40 and 45 rpm. During the constant speed rotarod experiment, mice were given three trials per day. The mean latency to fall from the rotarod (for the three trials at each speed level) was recorded.

### Stationary horizontal thin-rod test

The stationary horizontal thin-rod test was used to measure motor coordination and balance as previously reported [71]. Each male mouse was placed on the thin-rod (15 mm in diameter, 50 cm long and placed 40 cm high to discourage jumping). The thin-rod was flanked at both ends by a large acryl wall to prevent falling at the end of the thin-rod. The maximum observation time was 1 min. Mice were trained for three consecutive days, receiving four trials per day with a 1 h inter-trial interval. The mean latency to fall from the thin-rod was recorded.

### Step through-type passive avoidance (PA) test

The PA test was performed as described previously [72]. Briefly, the apparatus consisted of an illuminated compartment (light room) and a dark one (dark room) connected by a guillotine door. In the training trial, the male mouse was first placed into the light room and after 10 s, the partitioning door was opened. When the mouse entered the dark room, a 2 s footshock at 0.8 mA was given through the grid floor. Twenty-four hours after the training trial, the mouse was tested for retention time by being placed mice into the light room and the step through latency was measured (maximum latency, 600 s).

### Contextual and cued fear conditioning test

The contextual and cued fear conditioning test was performed in a soundproof behavioral apparatus (Muromachi Kikai, Japan). Male mice were placed in a conditioning chamber, with a plexiglas front, gray side- and back-walls and electrical grid floors, and allowed to move freely for 2 min. They then received three pairings of a tone (20 s, 4000 Hz, 80 dB) as a conditioned stimulus (CS), and a co-terminating footshock (2 s, 0.5 mA) as the unconditioned stimulus (US), with an inter-stimulus interval of 2 min. After the last footshock, the mice remained in the conditioning chamber for 2 min. Twenty-four hours after the conditioning, mice were tested for contextual memory in the same conditioning chamber for 3 min. The cued testing was performed in a triangular chamber, with a plexiglas front, black and white stripe side- and back-walls and flat floors covered by paper towels. Mice were allowed to move freely for 2 min, and then received a tone (120 s, 4000 Hz, 80 dB). All experiments were observed using a video tracking system (Muromachi Kikai, Japan) to record the moving and freezing behavior of the mice. Freezing was defined as complete resting, when a mouse does not move for more than 1 s [73]. The loss of retention activity was evaluated by the activity suppression ratio in the contextual testing using the activity levels during the first 2 min conditioning before a shock delivery and during the first 2 min contextual testing, according to the formula: activity_test_/(activity_train_ + activity_test_), as previously reported [31, 32]. Similarly, in the cue-dependent testing, the suppression ratio was calculated using the activity levels during the first 3 min cued testing before cue-exposure and during the last 3 min after the exposure.

### Open field (OF) test

In the OF test, locomotor activity was measured for 60 min in a square chamber (70 × 70 × 30 cm) with a video tracking system (Muromachi Kikai, Japan). Each mouse was placed in the corner of a square chamber. The test was illuminated at 100 lux. Moving time, distance travelled, and time spent in the center area were recorded.

### Cliff avoidance (CA) test

In the CA test, cliff avoidance and jumping were measured for 7 min using a round platform (a transparent plexiglas cylinder with a diameter of 13 cm and a height of 20 cm). Each male mouse was placed on the platform, and the jumping behavior was defined as positive performing, while falling from the top was defined as passive performing [34].

### Marble burying (MB) test

In the MB test, each male mouse was placed in an open plexiglas cage (28 × 45 × 20 cm) with a 5 cm layer of fine bedding material and 25 equally spaced glass marbles (17 mm in diameter). The number of buried marbles was counted after 30 min. When 2/3 of a marble was in the fine bedding material, it was defined as buried [52].

### Nest building (NB) test

In the NB test, each male mouse was placed into a clean cage containing fine wood-chip bedding material and single cotton batting Nestlets® nesting material (2.5 g of 5 cm squares, Ancare, NY, USA) approximately 1 h before the dark phase. The next morning, unused Nestlet pieces were weighed and nests were evaluated on a rating scale of 1–5 [38]. The threshold of unused Nestlet pieces was more than 0.1 g. The rating scale was defined as follows: 1, Nestlet not noticeably touched; 2, Nestlet partially shredded (50–90 % remaining intact); 3, Nestlet mostly shredded but no identified nest (<50 % of the Nestlet remaining intact); 4, Nestlet mostly shredded and a flat nest identified (>90 % of the Nestlet was shredded and used for the nest); and 5: almost all of the Nestlet was shredded and the nest was the shape of a crater with walls higher than mouse body height.

### Parity and maternal behavior

Female *Hbegf* cKO mouse was mated with a male *Hbegf*^*flox/flox*^ mouse. To rule out a possible influence of pups’ genetic background for maternal behavior, female *Hbegf*^*flox/flox*^ mouse was bred with male *Hbegf* cKO mouse as control mating. Pregnant mice were individually transferred to a new cage in the same environment and parturition was checked daily in the morning (09:00) and evening (19:00). The day when parturition was first detected was defined as postnatal day 0 (P0). Maternal behaviors were measured on P0 to P7. For the separation of a dam from her pups, the dam was put in one corner, while her pups were placed in the center of the same cage. After separation, maternal behavior, such as retrieving and suckling, were observed for 1 and 2 h. For this experiment, 10–12 weeks-old dams were used.

### BrdU- and doublecortin-staining

BrdU (50 mg/kg in sterile saline) was injected intraperitoneally [15]. Injections were made once a day for three consecutive days. For brain tissue preparation, mice were deeply anaesthetized with sodium pentobarbital (50 mg/kg, i.p.). Brains were quickly isolated, and washed with saline and 4 % PFA. Brains were post-fixed in 4 % PFA for 24 h and finally transferred to 25 % sucrose solution (in 0.1 M K^+^-free PBS) overnight for cryoprotection. Following freezing in cryoembedding compound, brain sections were prepared at 30 μm thickness and were stored in cryoprotectant solution (25 % ethylene glycol, 25 % glycerin, 0.05 M phosphate buffer) at −20 °C until processing for immunohistochemistry [16]. For the detection of BrdU-positive cells in brain sections, DNA denaturation was performed prior to incubation with anti-BrdU antibody as follows: incubation in 50 % formamide/2× SSC (0.3 M NaCl, 0.03 M sodium citrate) for 2 h at 65 °C, rinsing in 2× SSC for 5 min, incubation in 2 N HCl for 30 min at 37 °C, and rinsing in 0.1 M boric acid (pH 8.5) for 10 min at room temperature. Floating brain sections were incubated in 0.3 % H_2_O_2_ solution for 10 min, and rinsed with 0.1 % Triton X-100 in TBS (TBST). Brain sections were incubated with blocking buffer containing 3 % BSA and goat antiserum to mouse IgG (1:50; Cappel Laboratories, PA, USA) in TBST for 30 min. After thorough washing, the sections were reacted with mouse anti-BrdU monoclonal antibody (1:500: Roche, Basel, Switzerland), overnight at 4 °C. After further washing, the sections were incubated with secondary antibody, biotin-conjugated secondary antibody (1:500; goat polyclonal anti-mouse IgG, Invitrogen) for 2 h, and subsequently treated with avidin-biotin peroxidase solution (ABC kit, Vectastain Vector, USA) for 1 h at room temperature. BrdU-positive cells were visualized by incubation with a solution containing 0.02 % 3,3′-diaminobenzidine tetrahydrochloride (DAB) (Dojindo, Kumamoto, Japan) and 0.005 % H_2_O_2_ (Wako, Japan) in 0.05 M Tris–HCl buffer (pH 7.6), 1 % cobalt chloride (CoCl_2_) and 1 % nickel sulphate (NiSO_4_) solution until brown reaction products appeared. The brain sections were dehydrated through a series of ethanol solutions and xylene, and cover-slipped with Permount® (Fisher Scientific, Waltham, MA, USA).

BrdU-immunopositive cells in the olfactory bulb (OLF), rostral migratory stream (RMS), subventricular zone (SVZ), and subgranular zone (SGZ) were counted in every eighth section in a series of 30 μm brain sections using Image J software. The total number of BrdU-positive cells was estimated by multiplying the result by eight.

For the analysis of immature neuronal positioning, brain sections were incubated with guinea pig anti-doublecortin (DCX) polyclonal antibody (1:1000; Millipore, Billerica, MA, USA) overnight at 4 °C. After washing, the brain sections were labelled with second antibody, Alexa Fluor 594-conjugated anti-guinea pig IgG (1:1000), for 2 h at room temperature. Localization patterns of cells were evaluated using a previously described method with slight modifications [74]. Images of DCX-positive neurons with Hoechst 33342 staining were used to determine cell localization.

### Drug treatments

Atomoxetine hydrochloride was purchased as Strattera® Capsules from Eli Lilly, Indianapolis, IN, USA. The tablets were crushed and resuspended in physiological saline, and used for intraperitoneal administration (i.p.) at a dose of 1 mg/kg. D-cycloserine (Wako Chemical, Japan), a selective partial agonist of the NMDA receptor glycine recognition site, was dissolved in physiological saline, and used at 20 mg/kg (i.p.). Nefiracetam (*N*-[2,6-Dimethylphenyl]-2-[2-oxo-1-pyrrolidinyl]acetamide), a pyrrolidone derivative nootropic drug, which potentiates neural activity via an interaction with the NMDA receptor glycine recognition site, was kindly provided by Daiichi Pharmaceutical Co., Ltd. (Tokyo, Japan). Before administration, nefiracetam was dissolved in physiological saline, and used at 1 mg/kg (i.p.). Saline was used for control (vehicle) injections. Atomoxetine, D-cycloserine, and nefiracetam were administered once a day for seven consecutive days and behavioral tests were performed 24 h after the last administration.

### Statistical analysis

Statistical analyses were done by one-way analysis of variance (ANOVA) with the Tukey’s multiple comparison *post-hoc* test, two-way repeated measures ANOVA, Student’s *t* test or Kaplan–Meier survival plots and log-rank test using Graph Pad Prism 6 software. All results are expressed as means ± standard error of the mean (s.e.m.).

## Additional file

Additional file 1: Figure S1. In *Gng7* ^*wt/cre*^ and *Hbegf* ^*flox/flox*^ mice, used for mating to generate *Hbegf* cKO mice, lacZ positive cells were not observed in the dentate gyrus. (TIF 9882 kb)

## Abbreviations

ACSF: artificial cerebrospinal fluid
ADHD: attention deficit hyperactivity disorder
ANOVA: analysis of variance
CA: cliff avoidance
CS: conditioned stimulus
DAT: dopamine active transporter
DCX: doublecortin
DCX: doublecortin
DG: dentate gyrus
ERK: extracellular signal-regulated kinase
fEPSP: field excitatory post-synaptic potential
GCL: granule cell layer
Gng7: G protein γ subunit 7
HB-EGF: heparin-binding epidermal growth factor-like growth factor
KO: knockout
LTP: long-term potentiation
MB: marble-burying
NB: nest building
NET: norepinephrine transporter
OCD: obsessive-compulsive disorder
OF: open-field
OLF: olfactory bulb
PFA: paraformaldehyde
PSA-NCAM: polysialylated neural cell adhesion molecule
PSD: postsynaptic density
qPCR: quantitative real-time polymerase chain reaction
RMS: rostral migratory stream
s.e.m.: standard error of the mean
SGZ: subgranular zone
SHR: spontaneously hypertensive rats
SNAP-25: synaptosomal-associated protein 25
ST: step-through type passive avoidance
SVZ: subventricular zone
US: unconditioned stimulus
