# Supplementary figures and images for: NMDA receptor agonists reverse impaired psychomotor and cognitive functions associated with hippocampal Hbegf-deficiency in mice
Source: Mol Brain. 2015 Dec 4;8:83. doi: 10.1186/s13041-015-0176-0 (PMC4670538; doi:10.1186/s13041-015-0176-0)

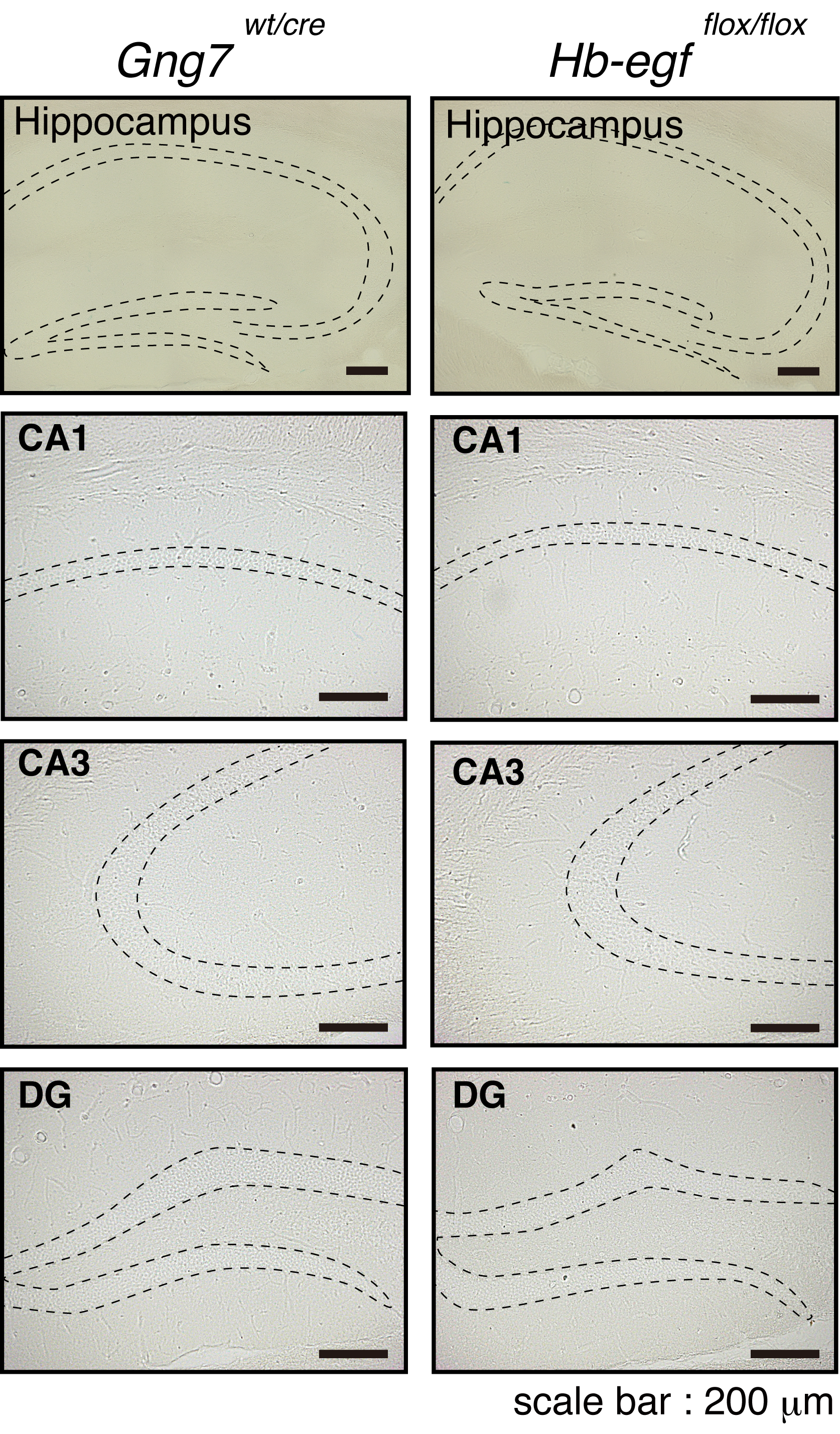

Supplement: Additional file 1: Figure S1. — In Gng7 wt/cre and Hbegf flox/flox mice, used for mating to generate Hbegf cKO mice, lacZ positive cells were not observed in the dentate gyrus. (TIF 9882 kb) [file 13041_2015_176_MOESM1_ESM.tif]
